# Continuous glucose monitoring in para cyclists: An observational study

**DOI:** 10.1002/ejsc.12220

**Published:** 2024-11-25

**Authors:** Vera Weijer, Rob van der Werf, Myrthe van der Haijden, Asker Jeukendrup, Luc J. C. van Loon, Jan‐Willem van Dijk

**Affiliations:** ^1^ School of Sport and Exercise HAN University of Applied Sciences Nijmegen the Netherlands; ^2^ Department of Human Biology NUTRIM Maastricht University Medical Centre+ Maastricht the Netherlands; ^3^ School of Sport Exercise and Health Sciences Loughborough University Loughborough UK

**Keywords:** continuous glucose monitoring, exercise, glucose, hyperglycemia, hypoglycemia, paralympic, spinal cord injury

## Abstract

Continuous glucose monitoring (CGM) is an emerging tool for dietary counseling in athletes. This study aimed to explore blood glucose profiles in Para cyclists and evaluate CGM accuracy at rest and during exercise. Thirteen Para cyclists, comprising eight hand bikers and five cyclists, wore a CGM sensor (Abbott) for 2 weeks. Participants recorded the timing of meals and regular training sessions and executed one standardized training session. Fifteen capillary blood glucose reference values (seven at rest and eight during the standardized training) were obtained by finger pricks. Mean glucose concentrations and time spent in hypoglycemia (<3.9 mmol/L), euglycemia (3.9–7.8 mmol/L), and hyperglycemia (>7.8 mmol/L) were calculated over 24 hrs and during daytime, nighttime, exercise, and 2 hrs postprandial periods. Mean absolute relative differences (MARD) were calculated between the CGM and capillary blood glucose. The mean glucose concentration over the 24 hr‐period was 5.7 (5.6–5.8) mmol/L. Athletes were in the euglycemia range 91% of the time. Hyperglycemia was almost exclusively observed postprandially and during exercise. Hypoglycemia was restricted to the night and was particularly observed in athletes with a spinal cord injury. CGM accuracy was acceptable at rest (MARD: 12%) but markedly lower during exercise (MARD: 34%; *p* = 0.01), especially for hand bikers (MARD: 41%) compared with cyclists (MARD: 24%; *p* = 0.01). Para cyclists generally do not display signs of disturbed glucose regulation. However, the increased risk for nocturnal hypoglycemia in athletes with a spinal cord injury warrants attention. Furthermore, CGM accuracy is compromised during exercise, especially if the sensor is in proximity to highly active muscles.

## Highlights

- Para cyclists generally do not display signs of disturbed glucose regulation.
- Para cyclists with a spinal cord injury seem to be at a higher risk for nocturnal hypoglycemia.
- Accuracy of the continuous glucose monitor is acceptable at rest but low during exercise, especially if the sensor is placed in proximity to highly active muscles.

## 1 INTRODUCTION

A continuous glucose monitor (CGM) is a wearable device that automatically monitors blood glucose concentrations over 24 hrs at intervals ranging from 1 to 15 min, depending on the device. CGM devices were originally developed to assist in the blood glucose control in patients with diabetes, and their application has been well studied in this population (Hoeks et al., 2011). Over the past decade, CGM devices have been improved in accuracy, longevity, and comfort. Consequently, these devices have garnered interest in the sports community. It is generally believed that monitoring blood glucose concentrations in real time can potentially benefit athletes, coaches, and sport nutritionists in optimizing nutritional strategies (Bowler et al., 2022; Flockhart et al., 2023).

It has long been established that hypoglycemia has a negative effect on exercise performance. In this regard, the development of fatigue during prolonged exercise has been linked to hypoglycemia (Coyle, 1992), emphasizing the importance of preventing hypoglycemia during exercise. Besides hypoglycemia, hyperglycemia is also believed to detrimentally affect performance, potentially by impairing decision‐making, as observed in a case study of an IndyCar driver with type 1 diabetes (Ferguson et al., 2018). Chronic hyperglycemia has also been suggested to be a negative regulator of aerobic adaptations to exercise (MacDonald et al., 2020). Additionally, hyperglycemia may influence substrate selection during exercise (MacLaren et al., 1999), potentially augmenting carbohydrate oxidation and, as such, accelerating muscle and liver glycogen depletion.

Limited evidence suggests that elite athletes spend more time in both hypo‐ and hyperglycemia compared to healthy controls (Flockhart et al., 2021). In this context, hyperglycemia seems to occur mainly in the early afternoon, while hypoglycemia occurs mainly during the night. Nocturnal hypoglycemia can potentially disrupt sleep by triggering an awakening response (Jauch‐Chara et al., 2010), which decreases sleep efficiency and increases wakefulness (Gais et al., 2003), thereby potentially impacting physical and mental performance, increasing injury risk, and impeding recovery (Charest et al., 2022; Fullagar et al., 2015). Despite the potential impact of hypo‐ and hyperglycemia on performance and recovery, information on the prevalence of hypo‐ and hyperglycemia in athletes without diabetes is quite scarce (Flockhart et al., 2021; Skroce et al., 2024; Thomas et al., 2017), and fully absent in Paralympic athletes. Such information could be of particular interest for Paralympic athletes, especially those with spinal cord injuries (SCI), who might have impaired counter‐regulation to hypoglycemia due to autonomic dysfunction (Naftchi, 1985).

While the current generation CGM devices are generally accurate and reliable for assessing blood glucose concentrations in nondiabetic individuals (Akintola et al., 2015), their performance during exercise could be an issue of concern. Multiple studies have reported a reduced accuracy during exercise (Bauhaus et al., 2023; Clavel et al., 2022; Muñoz Fabra et al., 2021; Thomas et al., 2017), with some studies noting an absolute decrease in accuracy (Muñoz Fabra et al., 2021) or a delay (Bauhaus et al., 2023; Thomas et al., 2017) in CGM‐derived glucose concentrations when compared to blood glucose concentrations from blood samples collected, while others reporting satisfactory accuracy of CGM devices during high‐intensity intermittent exercise in people with type 1 diabetes (Bally et al., 2016).

This study evaluates the blood glucose profiles of Para cyclists by examining the prevalence of hyper‐ and hypoglycemia, throughout the day and night, during exercise, and following meals. Furthermore, we evaluated the agreement in blood glucose concentrations obtained by the CGM and those obtained via capillary blood glucose sampling, both at rest and during exercise.

## 2 METHODS

### 2.1 Study design

In this cross‐sectional study, blood glucose concentrations of Para cyclists were monitored for up to 14 consecutive days. The athletes wore a CGM for a duration of 10–14 days, depending on sensor adhesion. During this period, athletes recorded the timing of their meals, as well as the timing and characteristics of their training sessions. In addition, one standardized training was executed to assess the agreement between CGM and capillary blood glucose concentrations during exercise. Capillary blood glucose samples were also taken in resting condition to assess the agreement between CGM and capillary glucose concentration at rest. The study was conducted between March and May 2023, and was approved by the Medical Ethical Committee Zuyd (NL82746.096.22).

### 2.2 Participants

A total of 16 Para cyclists (hand bikers, tandem cyclists, track and road‐race cyclists) competing at the highest (inter)national level were recruited through an ongoing partnership between the HAN University of Applied Sciences, the Royal Dutch cycling union (KNWU), and TeamNL. Exclusion criteria were pregnancy, an injury that disrupted the regular training regimen, or diagnosed diabetes mellitus. All participants were informed about the nature and potential risks of the study before providing their written informed consent, in accordance with the code of ethics of the Declaration of Helsinki.

### 2.3 Study protocol

**FIGURE 1:**
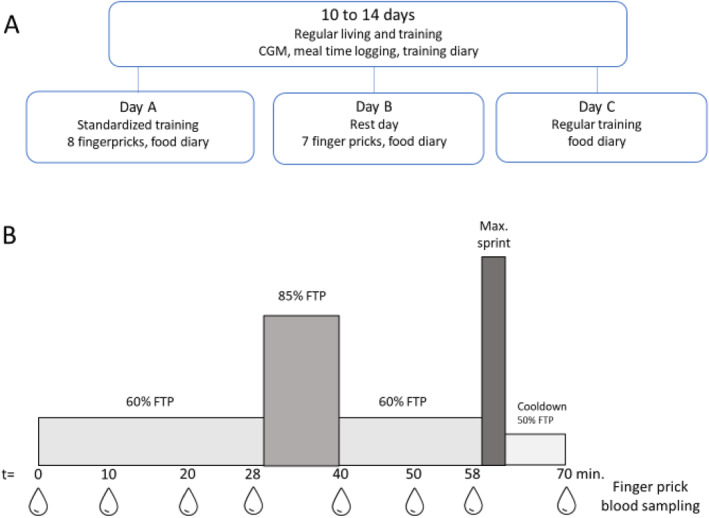
Study design. (A) Participants wore the CGM sensor for a 2‐week period (10–14 days), while logging the timing of their main meals, and kept a training log. Within the 2‐week period, three separate days were planned. On day A, participants performed a standardized exercise session and kept a detailed food record. Day B was scheduled on a rest day, during which the participants took seven capillary blood glucose samples at home and kept a detailed food record. During day C, which was scheduled on a regular training day, participants followed their normal training schedule and kept a detailed food record. (B) The standardized training of day A, consisted of (hand)biking for 70 min, starting at 60% of the functional threshold power (FTP) for 30 min, followed by 10 min at 85% FTP, 20 min at 60% FTP, a maximal sprint of 3 minutes, and a cooldown of 10 min at 50% FTP. During the standardized training, eight capillary blood glucose samples were taken by the researchers at *T* = 0, 10, 20, 28, 40, 50, 58, and 70.

The 14‐day period commenced with the application of the CGM sensor by a researcher according to the manufacturer's instruction. Based on the participants' training schedule, 3 days (day A, B, and C) within the 14‐day period were scheduled for a more detailed examination of glycemic control (Figure 1A). On day A, participants performed a standardized exercise session in an overnight fasted state and kept a detailed food record for the remainder of the day. Day B was scheduled on a rest day, during which the participants took seven capillary blood glucose samples at home and kept a detailed food record. During day C, which was scheduled on a regular training day, participants followed their normal training schedule and kept a detailed food record. Exercise training data were recorded digitally throughout the 14‐day period.

### 2.4 Continuous glucose monitoring

Glucose concentrations were measured using an Abbott Libre Sense Glucose Sport Biosensor (Abbott Laboratories, Chicago, IL, US). The sensor was placed on the posterior upper arm, as suggested by the manufacturer. The sensors are equipped with a 4 mm‐needle that penetrates the subcutaneous tissue. Within the interstitial fluid, the CGM sensor operates using a glucose oxidase‐soaked electrode at the needle. This enzyme, upon interacting with glucose, produces an electrical current, which is calibrated to reflect capillary blood glucose concentrations (Hoss et al., 2010). The predecessors of the Abbott Libre Sense Glucose Sport Biosensor, the Abbott Freestyle Libre and Abbott Freestyle Libre 2, reported a MARD of 11.4% and 9.2% in a diabetic population, respectively (Alva et al., 2022; Bailey et al., 2015). The minute‐to‐minute data from the sensor were accessed and recorded using Supersapiens application (TT1 Products Inc., Atlanta, GA, US), requiring a Bluetooth connection with an NFC‐enabled mobile phone. In case the Bluetooth connection was lost, data were temporarily stored on the sensor at 15‐min intervals. Participants could upload their data to the Supersapiens application by scanning the sensor with the smartphone's NFC chip within 8 hrs after the Bluetooth connection was lost.

### 2.5 Capillary blood samples

To assess accuracy, CGM‐derived glucose concentrations were compared with capillary blood glucose concentrations measured during a standardized exercise session and in resting conditions. The capillary blood was obtained by a finger prick at the fingertips, and glucose concentrations were measured with a Contour Next One glucose meter (Ascensia Diabetes Care, Parsippany, NJ) with corresponding test strips. The Contour Next glucose meter (the predecessor of the Contour Next One) has been reported as the most accurate self‐monitoring blood glucose device available, with a bias of −1.2% and a coefficient of variation (CV) of 5.3% (Klonoff et al., 2018). During the standardized exercise session, eight capillary blood samples were taken by a researcher on pre‐specified time points during the exercise session (Figure 1B). On a designated rest day (day B; Figure 1A), participants collected seven capillary blood samples at home. The samples were taken 5 min before and 1 hr after each main meal (breakfast, lunch, and dinner), as well as immediately before bedtime. During the study instruction, the researchers provided a demonstration of the finger‐pricking procedure, accompanied by written instructions. Participants were instructed to begin by carefully washing and drying their hands. They were then instructed to use the provided lancet device to puncture one of their fingers, discard the initial blood drop, apply the second drop onto the testing strip, and insert it into the Contour Next One device.

### 2.6 Nutritional intake

On three separate days (day A, B, and C; Figure 1A) during the 14‐day period, participants reported their full nutritional intake by means of a food record. Data on timing, products, and portion sizes were collected. The dietary records were processed using Compl‐eat software (Wageningen University, Division of Human Nutrition) to calculate the intake of energy, carbohydrates, mono‐ and disaccharides, protein, and fat for each main meal and over 24 hrs. On the days without a detailed food record, participants used the Supersapiens app to digitally log the timing of their main meals, that is, breakfast, lunch, and dinner. The 2‐hr postprandial phase after each main meal was analyzed for mean glucose, peak glucose, and time‐to‐peak glucose.

### 2.7 Exercise

On day A (Figure 1A), the participants performed a standardized exercise session in an overnight fasted state. The training session was scheduled to start between 9 and 11 a.m. and lasted 70 min. Training load was based on participants' self‐reported functional threshold power (FTP). Participants started with 30 min of cycling at 60% FTP, followed by a 10‐min block at 85% FTP, a 20‐min block at 60% FTP, a full 3‐min sprint, and a 10‐min cooldown period at 50% FTP (Figure 1B). During this exercise session, eight capillary blood glucose samples were taken by the researchers at *T* = 0, 10, 20, 28, 40, 50, 58, and 70 min. As capillary blood samples were collected from the fingertips, hand bikers were required to stop cycling for ∼30 s to obtain a blood sample.

The Para cyclists used TrainingPeaks software (NaN) to record all other training sessions throughout the 14‐day period. Participants were already familiar with this application. Data on duration, timing, type of exercise, and heart rate were collected. Heart rates were measured using participants' personal chest strap heart monitors. In addition, during cycling or hand bike sessions, workload was assessed using participants' personal crank power meters from either SRM (Schoberer Rad Meßtechnik, Jülich, Germany) or Quarq (Quarq, Spearfish, SD, USA). Data on workload were not collected during other exercise modes, such as wheeling, rowing, swimming, or strength training.

### 2.8 Data analysis

The research team had access to participants' CGM data through the online Supersapiens dashboard application (Supersapiens Dashboard). Raw data from this application were downloaded in the Excel format. Since the accuracy of CGMs is lower in the first day after application of the sensor (Zisser et al., 2009), the first 24 hrs of data were discarded. The Excel file was configured for automated analysis of glycemic outcomes using the GlyCulator 3.0 software (Chrzanowski et al., 2023), employing minute‐by‐minute data, equating to 1,440 data points over a 24‐hr period. Data gaps resulting from Bluetooth disconnection were filled using linear interpolation, but only for interruptions ≤30 min. Data gaps >30 consecutive min were excluded from the glycemic outcome analysis. Data were analyzed over 24 hrs, during daytime (6:00–23:59), nighttime (00:00–5:59), during exercise, and for all 2‐hr postprandial periods after the initiation of each main meal during the 14‐day period. The time spent in glucose zones was assessed by using the following cutoff points (Danne et al., 2017): severe hypoglycemia (<2.9 mmol/L), hypoglycemia (<3.9 mmol/L), euglycemia (3.9–7.8 mmol/L), hyperglycemia (>7.8 mmol/L), and severe hyperglycemia (>10.0 mmol/L). To calculate the glycemic variability, the coefficient of variation was calculated as the mean divided by the SD. The mean amplitude of glycemic excursions (MAGE) was calculated based on the arithmetic mean of differences between consecutive peaks and nadirs of differences greater than one SD of mean glycemia (Czerwoniuk et al., 2011).

To evaluate accuracy, CGM‐derived glucose concentrations were compared with capillary blood glucose concentrations. For this purpose, the timings of the capillary blood glucose measurements were synchronized with the corresponding CGM data, which were smoothed by averaging with the preceding and following three data points. The mean absolute relative difference (MARD) was calculated as follows: (capillary glucose—CGM glucose)/capillary glucose × 100%. Outliers in MARD were determined as values exceeding the third quartile plus 1.5 × interquartile range (Q3‐Q1), according to the box‐and‐whiskers plot method of Tukey (1977). Outliers of the MARD were calculated based on all available data points, but separately for exercise and rest. If a participant had more than two outliers (out of the seven data points for rest or eight data points for exercise), the participant was excluded for the analysis of accuracy.

Normally distributed data were expressed as mean ± SD, while non‐normally distributed data were expressed as median (Q1 to Q3). Differences in glycemic outcomes between day‐ and nighttime were analyzed with a paired samples *t*‐test for normally distributed data or a Wilcoxon signed ranks test for non‐normally distributed data. Differences in glycemic outcomes between breakfast, lunch, and dinner were analyzed with repeated measures ANOVA with Bonferroni correction for normally distributed data, while non‐normally distributed data were analyzed with a Friedman's test, with post hoc Wilcoxon signed ranks test with Bonferroni correction. Differences in glycemic parameters and MARD between subgroups were analyzed with an independent samples *t*‐test for normally distributed data and a Mann–Whitney *U* test for non‐normally distributed data.

## 3 RESULTS

### 3.1 Participants

Participants' characteristics are provided in Table 1. During data collection, three participants withdrew from the study: two due to issues with connection between the sensor and their mobile phone and one participant because of a lack of time and commitment. Of the remaining 13 participants, eight were hand bikers and five were cyclists (including tandem riders). Hand bikers either had a spinal cord injury or a limb deficiency, while cyclists had cerebral palsy, a visual impairment, nerve damage in the lower extremities, or no disability (pilot of a visual impaired athlete). Nine of the 13 athletes participated in the Paris Paralympic Games 2024 and won a total of 12 medals (6 gold, 3 silver, and 3 bronze). Furthermore, eight athletes were ranked within the top five in the 2024 world rankings in their respective categories.

**TABLE 1:** Participant characteristics.

	Total (*n* = 13)	Hand bikers (*n* = 8)	Cyclists (*n* = 5)
Disability (*n*)			
Spinal cord injury	4	4	0
Limb deficiency	4	4	0
Cerebral palsy	2	0	2
Visual impaired	1	0	1
Lower leg nerve damage	1	0	1
Nondisabled/pilot	1	0	1
Sex (male/female)	11/2	7/1	4/1
Age (years)	35 ± 8	36 ± 10	31 ± 6
Height (cm)	177.3 ± 21.7	171.3 ± 29.5	183.2 ± 10.2
Weight (kg)	72.0 (70.0–78.0)	72.0 (65.8–75.8)	73.0 (67.5–92.0)
Mean daily exercise duration (min/day)	120 ± 26	112 ± 28	133 ± 18
Type of exercise training (*n/athlete*)			
Endurance	8.9	8	10.4
Resistance	1.4	1.5	1.2
Other	0.9	1.5	0
Mean training workload endurance (kJ/session)	1194 ± 561	949 ± 396	1623 ± 593^a^
Functional threshold power (W)	260 ± 81	217 ± 68	328 ± 45^b^
Mean heart rate endurance exercise (bpm)	137 ± 11	136 ± 12	138 ± 9

*Note:* Data is presented as mean ± SD or median (IQR). Other training types included: wheeling, swimming, and rowing.

^a^ Significantly different between hand bikers and cyclists *p =* 0.048.

^b^ Significantly different between hand bikers and cyclists *p =* 0.008.

During the 14‐day period, most training sessions were hand biking or cycling sessions (79% of the training sessions), with some resistance training (12%) and other types of training (8%) in the form of wheeling, swimming, and rowing. Mean daily training durations were comparable between hand bikers and cyclists, although the absolute workload of the hand bike or cycling training sessions was significantly higher for the cyclists (*p* = 0.048). The median energy intake was 2768 (2354–3784) kcal/day, comprising 373 ± 130 g of carbohydrates, 143 (109–151) *g* of protein, and 96 (84–125) *g* of fat. Further dietary intake data for the main meals are provided in Table S1 in Supporting Information S1.

### 3.2 Glycemia over 24 hrs and during daytime and nighttime

**FIGURE 2:**
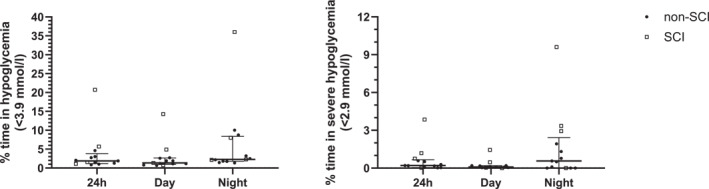
Percentage of time spent in hypoglycemia (<3.9 mmol/L) and severe hypoglycemia (<2.9 mmol/L) during 24 hrs, daytime, and nighttime, in participants with and without spinal cord injury as measured with continuous glucose monitoring. Data are presented as median ± IQR.

A full overview of glycemia over 24 hrs and during daytime and nighttime is provided in Table 2. The median of mean 24 hrs glucose concentrations was 5.7 (5.6–5.8) mmol/L and was higher during daytime (5.9 (5.7–6.0) mmol/L) compared with nighttime (4.9 (4.9–5.3) mmol/L; *p* = 0.001; Table 2). Overall, participants were in the euglycemic range (3.9–7.8 mmol/L) for 91% (90%–94%) of the time. This percentage was higher during nighttime (97% (89%–97%)) compared with daytime (90% (89%–92%); *p* = 0.046). This can be ascribed to the higher prevalence of hyperglycemia during daytime (7% (6%–10%)) compared with nighttime (0.2% (0.1%–0.2%); *p* = 0.001). Severe hyperglycemia (>10 mmol/L) was uncommon, with a median duration of 0.2% (0.1%–0.5%) of the 24‐hr period. None of the participants spent any time in severe hyperglycemia during the night. At the other end of the spectrum, 2.1% (1.6%–3.6%) of the 24 hr‐period was spent in hypoglycemia (<3.9 mmol/L). The relative prevalence of hypoglycemia was higher during nighttime 3.2% (2.4%–10.5%) compared with daytime (1.4% (1.2%–2.7%) *p* = 0.001). Figure 2 shows that hypoglycemia and severe hypoglycemia during the night were mainly observed in the athletes with SCI (8.2% (3.0%–37%) and 3.1% (0.7%–8.0%) of the time, respectively). One of the athletes with SCI even spent 46% of the nighttime in hypoglycemia, with 9.6% of the time being spent in severe hypoglycemia.

**TABLE 2:** Markers of glycemia.

	24‐hr period	Daytime	Nighttime	*p*‐value
Mean glucose (mmol/L)	5.7 (5.6–5.8)	5.9 (5.7–6.0)	4.9 (4.9–5.3)	**0.001**
<2.9 mmol/L (%time)	0.2 (0.1–0.6)	0.1 (0.0–0.2)	0.6 (0.0–1.9)	**0.033**
<3.9 mmol/L (%time)	2.1 (1.6–3.6)	1.4 (1.2–2.7)	3.2 (2.4–10.5)	**0.001**
3.9–7.8 mmol/L (%time)	90.8 (89.8–93.7)	89.9 (88.5–92.4)	96.5 (89.5–97.3)	**0.046**
>7.8 mmol/L (%time)	5.4 (4.5–7.2)	7.4 (6.0–9.5)	0.2 (0.1–0.2)	**0.001**
>10.0 mmol/L (%time)	0.2 (0.1–0.5)	0.3 (0.1–0.7)	0.0 (0.0–0.0)	**0.002**
MAGE	1.9 ± 0.2	2.0 ± 0.2	1.3 ± 0.2	**<0.001**
CV	20.6 ± 2.6	20.1 ± 2.3	14.8 ± 2.7	**<0.001**
SD	1.2 ± 0.1	1.2 ± 0.1	0.7 ± 0.1	**<0.001**

*Note*: Data are presented as mean ± SD or median (IQR). *P*‐value is reported for difference between day‐ and nighttime, bold values represent a significant difference (*p* < 0.05).

### 3.3 Glycemia during exercise

The mean glucose concentration during exercise was 6.3 ± 0.4 mmol/L, with the participants spending 12.7% (8.1%–33.5%) of the time in (mild) hyperglycemia. However, severe hyperglycemia was uncommon, with a median duration of 0.7% (0.0%–2.2%) of the time during exercise. Hypoglycemia was nearly absent during exercise with 0.2 (0.0–1.6)% of the time, while severe hypoglycemia was completely absent.

### 3.4 Postprandial glycemia

Markers of postprandial glycemia are shown in Table 3. The mean glucose concentrations were significantly higher following lunch compared with breakfast (*p* = 0.003), while the time spent in hyperglycemia was significantly higher after lunch compared with dinner (*p* = 0.040). Mild hyperglycemia (>7.8 mmol/L) was relatively common in the postprandial phase, whereas severe hyperglycemia (>10 mmol/L) barely occurred. Additionally, mild hypoglycemia (<3.9 mmol/L) had a low prevalence in the postprandial phase, while severe hypoglycemia (<2.9 mmol/L) was completely absent.

**TABLE 3:** Markers of postprandial glycemia.

	Breakfast	Lunch	Dinner	*P*‐value
Mean glucose (mmol/L)	6.0 ± 0.5	6.4 ± 0.6	6.1 ± 0.4	**0.004** ^a^
<2.9 mmol/L (%time)	0.0 (0.0–0.0)	0.0 (0.0–0.0)	0.0 (0.0–0.0)	N.A.
<3.9 mmol/L (%time)	1.3 (0.0–3.3)	0.6 (0.0–2.3)	1.2 (0.0–3.6)	0.442
3.9–7.8 mmol/L (%time)	86.1 (79.7–94.1)	81.3 (74.4–90.0)	90.0 (83.5–96.5)	0.116
>7.8 mmol/L (%time)	7.6 (4.2–14.7)	15.7 (8.7–22.1)	5.9 (2.8–14.5)	**0.037** ^b^
>10.0 mmol/L (%time)	0.0 (0.0–0.4)	0.0 (0.0–2.2)	0.0 (0.0–0.4)	0.748
MAGE	1.9 ± 0.5	2.0 ± 0.5	1.6 ± 0.7	0.210
CV	5.8 ± 0.6	6.4 ± 0.6	6.0 ± 0.5	**0.003** ^a^
SD	1.0 ± 0.3	1.0 ± 0.2	0.9 ± 0.2	0.322
Individual mean peak glucose (mmol/L)	8.1 ± 0.9	8.4 ± 0.8	7.9 ± 0.7	0.179
Time to peak (min)	46 ± 8	52 ± 14	49 ± 16	0.584

*Note*: Data are presented as mean ± SD or median (IQR). *P*‐values of the main effect are presented, bold values represent a significant effect (*p* < 0.05). N.A.: statistical test could not be performed because of absence of severe hypoglycemia in the postprandial periods.

^a^ Significant difference between breakfast and lunch.

^b^ Significant difference between lunch and dinner.

### 3.5 CGM versus capillary blood glucose concentrations

**FIGURE 3:**
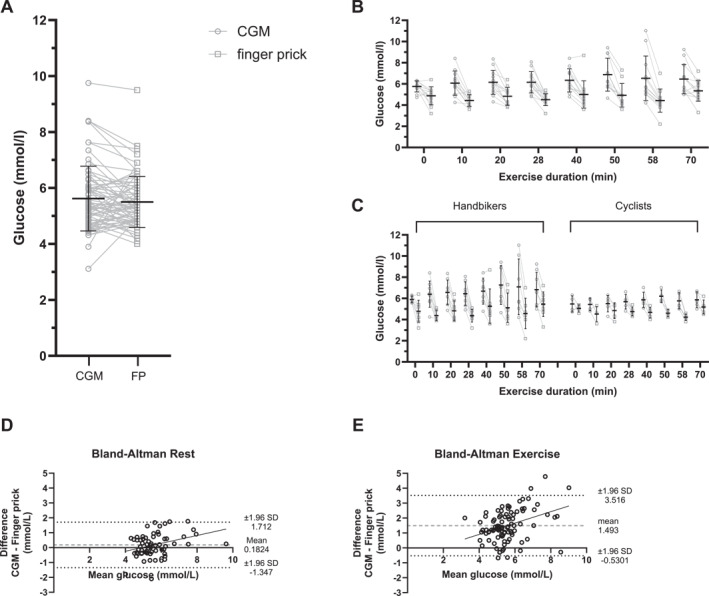
Accuracy of CGM during resting and exercise conditions. Blood glucose concentrations of corresponding CGM and capillary measurements assessed during resting conditions (A) and over the time during standardized exercise (B). (C) blood glucose concentrations during exercise are shown for hand bikers and cyclists separately. (D) and (E) represent the Bland–Altman plots of the blood glucose values measured with the CGM and finger prick method during rest (D) and during exercise (E). Data are presented as mean ± SD.

During resting conditions, four (out of 91) matched data points were missing across three participants, attributed to the absence of capillary recordings from these individuals. As shown in Figure 3A, the mean glucose concentration assessed over all time points was comparable between capillary blood samples (5.5 ± 0.9 mmol/L) and corresponding CGM‐derived samples (5.6 ± 1.2 mmol/L; *p* = 0.484). The MARD was 12 ± 6%, with the Bland–Altman analysis (Figure 3D) showing a mean bias of 0.2 ± 0.8 mmol/L with limits of agreement ranging from −1.3 to 1.7 mmol/L. During exercise, seven (out of 104) matched data points were missing across four participants, two due to unsuccessful finger pricks and five due to missing CGM data recordings. The mean capillary blood glucose concentration during the exercise session in the fasted state was 4.8 ± 1.0 mmol/L, while the mean blood glucose concentration of corresponding CGM measurements was 6.3 ± 1.3 mmol/L (*p* < 0.001; Figure 3B). The Bland–Altman analysis (Figure 3E) revealed a mean bias of 1.5 ± 1.0 mmol/L with limits of agreement ranging from −0.5 to 3.5 mmol/L. This discrepancy was accompanied by a MARD of 34 ± 12%, which was significantly higher compared to the MARD in resting conditions (*p* < 0.001). Moreover, while capillary blood glucose concentrations remained relatively stable during the 60‐min exercise session, CGM‐derived blood glucose concentrations appeared to rise, leading to an increasing discrepancy between capillary and CGM‐derived glucose concentrations over the course of the exercise session. It is interesting to note that the MARD during exercise was substantially higher in hand bikers (41 ± 9%) compared with cyclists (24 ± 11%; *p* = 0.01; Figure 3C).

## 4 DISCUSSION

The current study employed CGM to assess 24‐hr glycemic control in elite Para cyclists and assessed the accuracy of CGM in both resting and exercise conditions. The study revealed that over the 24‐hr period, athletes are mainly in euglycemia. Some mild hyperglycemic periods (>7.8 mmol/L) were noted during the daytime, mostly following meals and during exercise, whereas more severe hyperglycemia (>10 mmol/L) was uncommon. Hypoglycemia (<3.9 mmol/L) occurred more frequently at night compared with daytime, although its extent was limited in most athletes. Nevertheless, athletes with SCI seemed to have a higher risk of severe nocturnal hypoglycemia (<2.9 mmol/L). Furthermore, the accuracy of the CGM appeared satisfactory under resting conditions but became compromised during exercise, especially in the case of hand bikers.

### 4.1 Time spent in glycemic ranges

The elite level Para cyclists in our cohort spent 91% of the time in the euglycemic range, which is comparable to the 89% found in elite athletes without a disability (Flockhart et al., 2021) and the 93% in a large cohort of healthy active individuals (Skroce et al., 2024). This large proportion of time spent in euglycemia aligns with the expectations of healthy individuals without diabetes, where blood glucose homeostasis is tightly regulated (Sprague et al., 2011). Nevertheless, time spent in euglycemia was somewhat lower than the 96% in a healthy nonathletic population (Shah et al., 2019). This small discrepancy can be ascribed to the additional time our participants spent in hyperglycemia. Given the mean training duration of 2 hrs per day, alongside the relative increase of hyperglycemia during exercise, the exercise itself appears to be an important factor explaining the variance in time spent outside the euglycemic range. Furthermore, the greater energy expenditure in athletes compared with nonathletes necessitates a higher energy intake. This can be achieved by consuming larger meals, which may provoke a greater postprandial glycemic response. Indeed, the athletes in our cohort spent more than 1 hr a day (5.4% of the time) in hyperglycemia, which is marginally higher than the 2.4%–3.6% reported in healthy nonathletic populations (Shah et al., 2019; Skroce et al., 2024). Although chronic hyperglycemia is a potential negative regulator of aerobic adaptations to exercise (MacDonald et al., 2020) and therefore potentially negative for endurance athletes, the minor and mild hyperglycemic episodes observed in the current study are unlikely to hamper endurance exercise adaptations. Indeed, acute hyperglycemia does not affect muscular strength, power, or endurance performance in healthy active individuals (Lime‐Ma et al., 2017). However, whether the mild hyperglycemic episodes during exercise observed in the current study affect substrate utilization, as previously reported with hyperglycemic clamping during exercise, remains to be established (MacLaren et al., 1999).

The median time spent in hypoglycemia in the current study (2.1%) was comparable to Swedish swimmers (2.6%) (Olsson, 2017) and healthy active individuals (3.4%) (Skroce et al., 2024), but noticeably lower than the 8% reported in elite endurance athletes by Flockhart and coworkers (Flockhart et al., 2021), although the cutoff point for hypoglycemia was set marginally higher in the latter study. In line with the study of Flockhart et al., hypoglycemic episodes were mainly prevalent during the night, although there were substantial variations between individuals in the severity and duration of hypoglycemia. In this regard, particularly athletes with SCI seemed to be susceptible for nocturnal hypoglycemia. This may be attributed to a diminished counterregulatory response to hypoglycemia in individuals with SCI, stemming from dysregulation of the autonomic nervous system (Naftchi, 1985). One participant with SCI even spent almost half (46%) of his nights in hypoglycemia. Although severe nocturnal hypoglycemia has been demonstrated to induce an awakening response in healthy individuals (Banarer et al., 2003), it remains unclear whether individuals with SCI experience similar effects on sleep or exhibit an impaired epinephrine and neurogenic response like individuals with diabetes (Banarer et al., 2003). Furthermore, the implications of hypoglycemia‐induced impairments in sleep quality for recovery and performance have yet to be determined.

In terms of accuracy, the MARD between capillary and CGM‐derived glucose concentration in resting conditions (12%) was comparable with the reported MARD of the Abbott Freestyle Libre and Abbott Freestyle Libre 2 (11.4% and 9.2%, respectively) in individuals with diabetes (Alva et al., 2022; Bailey et al., 2015). Moreover, the mean bias between capillary and CGM‐derived glucose concentrations was only 0.2 mmol/L, with acceptable limits of agreement. Hence, the accuracy of CGM during resting conditions can be considered satisfactory. In contrast, a large discrepancy between CGM and capillary glucose concentrations was noted during exercise (MARD 34%), with a positive bias of 1.5 mmol/L with CGM compared with capillary glucose concentrations. The diminished accuracy of CGM during exercise in athletes without diabetes has also been recently shown by Clavel et al. (MARD 13%) (Clavel et al., 2022) and Bauhaus et al. (MARD 18%–22%) (Bauhaus et al., 2023). However, the MARD during exercise in the current study (34%) was markedly higher compared to previous reports. This discrepancy seems to be mainly attributed to the higher MARD in the hand bikers (41%) compared with the cyclists (24%) in the current study. When considering the MARD observed in the cyclists alone, results of the current study were in line with the MARD during moderate‐intensity cycling exercise (22%) as reported by Bauhaus and coworkers (Bauhaus et al., 2023). Moreover, while exercise intensity could play a role in the accuracy of CGM during exercise (Bauhaus et al., 2023), the current study suggests that exercise duration can also affect accuracy. While capillary blood glucose concentrations remained relatively stable during exercise in the fasted state, CGM‐derived glucose concentrations increased during exercise, resulting in an increasing discrepancy over the course of the exercise session. In this regard, the longer duration of the exercise session in the current study (70 min) compared with Clavel et al. (38 min) and Bauhaus et al. (60 min) may have contributed to the lower accuracy observed in the current study compared with both previous studies. Another difference between the studies is that exercise was performed in a fasted state in the current study, whereas participants in both previous studies exercised in a fed state.

Several mechanisms may contribute to the impaired accuracy of the CGM during exercise. Firstly, CGM sensors react with glucose in the interstitial fluid, where the resulting electrical current is calibrated to reflect capillary blood glucose concentrations (Hoss et al., 2010). Although glucose concentrations in the subcutaneous interstitial fluid and capillary blood are generally closely correlated, both exercise and the associated rise in temperature increase the blood flow and glucose flux in active muscle (Coates et al., 2023; Wasserman, 1995), which likely alters the ratio between local interstitial glucose concentrations and capillary blood glucose concentrations, leading to a reduced CGM accuracy (Moser et al., 2018). Furthermore, changes in temperature (Ricci et al., 2007) and pH (Bankar et al., 2009) induced by exercise potentially affect the accuracy of CGM by influencing the enzymatic activity of glucose oxidase. Altogether, these factors may also explain the markedly lower accuracy during exercise in hand bikers versus cyclists as observed in the current study. In both groups, the CGM sensors were positioned on the backside of the upper arm. It is evident that the upper arm muscles are more actively engaged in hand biking compared to cycling, which could lead to a higher blood flow, glucose flux, and temperature, and lower pH in the interstitial space surrounding the CGM sensor. Irrespective of the reasons for inaccurate glucose measurements during exercise, the positive bias of CGM‐derived glucose concentrations could result in an overestimation of the time spent in hyperglycemia in athletes. Consequently, the greater prevalence of hyperglycemia in elite athletes compared with healthy nonathletes, as reported by Flockhart et al. (2021), may not necessarily reflect an impaired glucose tolerance, but rather be an artifact due to inaccurate glucose readings during exercise.

### 4.2 Limitations

Some limitations of the current study are important to discuss. Firstly, accuracy of the CGM was assessed against capillary blood glucose concentrations measured by handheld capillary blood glucose monitors. Although the capillary blood glucose monitors used in the current study have been shown to be highly accurate (Klonoff et al., 2018), laboratory methods such as photo‐ or mass spectrometry, which are generally regarded as the gold standard for the assessment of glucose concentrations, could have provided even greater accuracy. Consequently, the reported MARD could be slightly over‐ or underestimated. It should also be noted that lying on the sensor during sleep can occasionally result in inaccurate glucose readings, likely due to restricted blood flow from tissue compression (Mensh et al., 2013). Although this issue has not been investigated for sensors placed on the posterior upper arm, the possibility of false positive nocturnal hypoglycemic readings cannot be ruled out in the current study. Finally, this study was conducted with a relatively small sample size, particularly in the context of subgroup analyses (i.e., SCI vs. non‐SCI and hand bikers vs. cyclists), potentially resulting in insufficient statistical power for certain subgroup analyses. Despite this limitation, the subgroup analyses highlight important areas for future investigation, specifically regarding the influence of SCI on blood glucose regulation in Para athletes, and the effect of sensor location combined with the type of exercise on the accuracy of CGM during exercise.

## 5 CONCLUSIONS

This study demonstrates that blood glucose concentrations of Para cyclists reside within the euglycemic range most of the time. Mild hyperglycemic (>7.8 mmol/L) episodes are commonly experienced by Para cyclists, particularly following meals and during exercise, while severe hyperglycemia (>10 mmol/L) is rarely observed. Mild hypoglycemia (<3.9 mmol/L) occasionally occurs during the night, while severe hypoglycemia (<2.9 mmol/L) is almost exclusively observed in athletes with an SCI, thus warranting attention in this population. While the accuracy of CGM is satisfactory at rest, discrepancies with capillary blood glucose sampling arise during exercise, particularly in hand bikers where CGM sensors were placed in proximity to highly engaged muscle. This underscores the need for caution in interpreting CGM‐derived glucose concentrations during exercise.

## CONFLICT OF INTEREST STATEMENT

No conflicts of interest, financial or otherwise, are declared by the authors. The results of this study are presented clearly, honestly, and without fabrication, falsification, or inappropriate data manipulation.

## Supporting information

Table S1

## Data Availability

The data that support the findings of this study are available from the corresponding author upon reasonable request.

## References

[ejsc12220-bib-0001] Akintola, Abimbola A. , Raymond Noordam , Steffy W. Jansen , Anton J. de Craen , Bart E. Ballieux , Christa M. Cobbaert , Simon P. Mooijaart , Hanno Pijl , Rudi G. Westendorp , and Diana van Heemst . 2015. “Accuracy of Continuous Glucose Monitoring Measurements in Normo‐Glycemic Individuals.” PLoS One 10(10): e0139973. 10.1371/journal.pone.0139973. 26445499 PMC4596806

[ejsc12220-bib-0002] Alva, Shridhara , Timothy Bailey , Ronald Brazg , Erwin S. Budiman , Kristin Castorino , Mark P. Christiansen , Gregory Forlenza , Mark Kipnes , David R. Liljenquist , and Hanqing Liu . 2022. “Accuracy of a 14‐day Factory‐Calibrated Continuous Glucose Monitoring System with Advanced Algorithm in Pediatric and Adult Population with Diabetes.” Journal of Diabetes Science and Technology 16(1): 70–77. 10.1177/1932296820958754. 32954812 PMC8875061

[ejsc12220-bib-0003] Bailey, Timothy , Bruce W. Bode , Mark P. Christiansen , Leslie J. Klaff , and Shridhara Alva . 2015. “The Performance and Usability of a Factory‐Calibrated Flash Glucose Monitoring System.” Diabetes Technology and Therapeutics 17(11): 787–794. 10.1089/dia.2014.0378. 26171659 PMC4649725

[ejsc12220-bib-0004] Bally, Lia , Thomas Zueger , Nicola Pasi , Ciller Carlos , Daniela Paganini , and Christoph Stettler . 2016. “Accuracy of Continuous Glucose Monitoring during Differing Exercise Conditions.” Diabetes Research and Clinical Practice 112: 1–5. 10.1016/j.diabres.2015.11.012. 26739116

[ejsc12220-bib-0005] Banarer, Salomon , and Philip E. Cryer . 2003. “Sleep‐related Hypoglycemia‐Associated Autonomic Failure in Type 1 Diabetes: Reduced Awakening from Sleep during Hypoglycemia.” Diabetes 52(5): 1195–1203. 10.2337/diabetes.52.5.1195. 12716752

[ejsc12220-bib-0006] Bankar, Sandip B. , Mahesh V. Bule , Rekha S. Singhal , and Laxmi Ananthanarayan . 2009. “Glucose Oxidase—An Overview.” Biotechnology Advances 27(4): 489–501. 10.1016/j.biotechadv.2009.04.003. 19374943

[ejsc12220-bib-0007] Bauhaus, Helen , Pinar Erdogan , Hans Braun , and Mario Thevis . 2023. “Continuous Glucose Monitoring (CGM) in Sports: A Comparison between a CGM Device and Lab‐Based Glucose Analyser under Resting and Exercising Conditions in Athletes.” International Journal of Environmental Research and Public Health 20(15): 6440. 10.3390/ijerph20156440. 37568982 PMC10418731

[ejsc12220-bib-0008] Bowler, A.‐Lee M. , Jamie Whitfield , Lachlan Marshall , Vernon G. Coffey , Louise M. Burke , and Gregory R. Cox . 2022. “The Use of Continuous Glucose Monitors in Sport: Possible Applications and Considerations.” International Journal of Sport Nutrition and Exercise Metabolism 33(2): 121–132. 10.1123/ijsnem.2022-0139. 36572039

[ejsc12220-bib-0009] Charest, Jonathan , and Michael A. Grandner . 2022. “Sleep and Athletic Performance: Impacts on Physical Performance, Mental Performance, Injury Risk and Recovery, and Mental Health: an Update.” Sleep Medicine Clinics 17(2): 263–282. 10.1016/j.jsmc.2022.03.006. 35659079

[ejsc12220-bib-0010] Chrzanowski, Jędrzej , Szymon Grabia , Arkadiusz Michalak , Anna Wielgus , Julia Wykrota , Beata Mianowska , Agnieszka Szadkowska , and Wojciech Fendler . 2023. “GlyCulator 3.0: A Fast, Easy‐To‐Use Analytical Tool for CGM Data Analysis, Aggregation, Center Benchmarking, and Data Sharing.” Diabetes Care 46(1): e3–e5. 10.2337/dc22-0534. 36356162 PMC9918444

[ejsc12220-bib-0011] Clavel, Pauline , Eve Tiollier , Cédric Leduc , Marina Fabre , Mathieu Lacome , and Martin Buchheit . 2022. “Concurrent Validity of a Continuous Glucose‐Monitoring System at Rest and during and Following a High‐Intensity Interval Training Session.” International Journal of Sports Physiology and Performance 17(4): 627–633. 10.1123/ijspp.2021-0222. 35193110

[ejsc12220-bib-0012] Coates, Alexandra M. , Jeremy N. Cohen , and Jamie F. Burr . 2023. “Investigating Sensor Location on the Effectiveness of Continuous Glucose Monitoring during Exercise in a Non‐diabetic Population.” European Journal of Sport Science 23(10): 2109–2117. 10.1080/17461391.2023.2174452. 36715137

[ejsc12220-bib-0013] Coyle, Edward F. 1992. “Carbohydrate Supplementation during Exercise.” The Journal of Nutrition 122(suppl_3): 788–795. 10.1093/jn/122.suppl_3.788. 1542049

[ejsc12220-bib-0014] Czerwoniuk, Dorota , Wojciech Fendler , Lukasz Walenciak , and Wojciech Mlynarski . 2011. “GlyCulator: a Glycemic Variability Calculation Tool for Continuous Glucose Monitoring Data.” Journal of Diabetes Science and Technology 5(2): 447–451. 10.1177/193229681100500236. 21527118 PMC3125941

[ejsc12220-bib-0015] Danne, Thomas , Revital Nimri , Tadej Battelino , Richard M. Bergenstal , Kelly L. Close , J. Hans DeVries , Satish Garg , et al. 2017. “International Consensus on Use of Continuous Glucose Monitoring.” Diabetes Care 40(12): 1631–1640. 10.2337/dc17-1600. 29162583 PMC6467165

[ejsc12220-bib-0016] Ferguson, David P. , and Nicholas D. Myers . 2018. “Physical Fitness and Blood Glucose Influence Performance in IndyCar Racing.” The Journal of Strength and Conditioning Research 32(11): 3193–3206. 10.1519/jsc.0000000000002879. 30239455

[ejsc12220-bib-0017] Flockhart, Mikael , and Filip J. Larsen . 2023. “Continuous Glucose Monitoring in Endurance Athletes: Interpretation and Relevance of Measurements for Improving Performance and Health.” Sports Medicine 54(2): 1–9. 10.1007/s40279-023-01910-4. 37658967 PMC10933193

[ejsc12220-bib-0018] Flockhart, Mikael , Lina C. Nilsson , Senna Tais , Björn Ekblom , William Apró , and Filip J. Larsen . 2021. “Excessive Exercise Training Causes Mitochondrial Functional Impairment and Decreases Glucose Tolerance in Healthy Volunteers.” Cell Metabolism 33(5): 957–970. 10.1016/j.cmet.2021.02.017. 33740420

[ejsc12220-bib-0019] Fullagar, Hugh H. K. , Sabrina Skorski , Rob Duffield , Daniel Hammes , Aaron J. Coutts , and Tim Meyer . 2015. “Sleep and Athletic Performance: the Effects of Sleep Loss on Exercise Performance, and Physiological and Cognitive Responses to Exercise.” Sports Medicine 45(2): 161–186. 10.1007/s40279-014-0260-0. 25315456

[ejsc12220-bib-0020] Gais, Steffen , Jan Born , Achim Peters , Bernd Schultes , Britta Heindl , Horst L. Fehm , and Werner Kern . 2003. “Hypoglycemia Counterregulation during Sleep.” Sleep 26(1): 55–59. 10.1093/sleep/26.1.55. 12627733

[ejsc12220-bib-0021] Hoeks, L. B. E. A. , W. L. Greven , and H. W. de Valk . 2011. “Real‐time Continuous Glucose Monitoring System for Treatment of Diabetes: a Systematic Review.” Diabetic Medicine 28(4): 386–394. 10.1111/j.1464-5491.2010.03177.x. 21392060

[ejsc12220-bib-0022] Hoss, Udo , Iman Jeddi , Mark Schulz , Erwin Budiman , Claire Bhogal , and Geoffrey McGarraugh . 2010. “Continuous Glucose Monitoring in Subcutaneous Tissue Using Factory‐Calibrated Sensors: a Pilot Study.” Diabetes Technology and Therapeutics 12(8): 591–597. 10.1089/dia.2010.0051. 20615099

[ejsc12220-bib-0023] Jauch‐Chara, Kamila , and Bernd Schultes . 2010. “Sleep and the Response to Hypoglycaemia.” Best Practice and Research Clinical Endocrinology and Metabolism 24(5): 801–815. 10.1016/j.beem.2010.07.006. 21112027

[ejsc12220-bib-0024] Klonoff, David C. , Joan Lee Parkes , Boris P. Kovatchev , David Kerr , Wendy C. Bevier , Ronald L. Brazg , Mark Christiansen , Timothy S. Bailey , James H. Nichols , and Michael A. Kohn . 2018. “Investigation of the Accuracy of 18 Marketed Blood Glucose Monitors.” Diabetes Care 41(8): 1681–1688. 10.2337/dc17-1960. 29898901

[ejsc12220-bib-0025] Lime‐Ma, Franklin , Joshua A. Cotter , and Evan E. Schick . 2017. “The Effect of Acute Hyperglycemia on Muscular Strength, Power and Endurance.” International Journal of Exercise Science 10(3): 390–396. 10.70252/sdbw3813. 28515835 PMC5421977

[ejsc12220-bib-0026] MacDonald, Tara L. , Pattarawan Pattamaprapanont , Prerana Pathak , Natalie Fernandez , Ellen C. Freitas , Samar Hafida , Joanna Mitri , Steven L. Britton , Lauren G. Koch , and Sarah J. Lessard . 2020. “Hyperglycaemia Is Associated with Impaired Muscle Signalling and Aerobic Adaptation to Exercise.” Nature Metabolism 2(9): 902–917. 10.1038/s42255-020-0240-7. PMC8278496 32694831

[ejsc12220-bib-0027] MacLaren, D. P. M. , T. Reilly , I. T. Campbell , and C. Hopkin . 1999. “Hormonal and Metabolic Responses to Maintained Hyperglycemia during Prolonged Exercise.” Journal of Applied Physiology 87(1): 124–131. 10.1152/jappl.1999.87.1.124. 10409566

[ejsc12220-bib-0028] Mensh, Brett D. , Natalie A. Wisniewski , Brian M. Neil , and Daniel R. Burnett . 2013. “Susceptibility of Interstitial Continuous Glucose Monitor Performance to Sleeping Position.” Journal of Diabetes Science and Technology 7(4): 863–870. 10.1177/193229681300700408. 23911167 PMC3879750

[ejsc12220-bib-0029] Moser, Othmar , Jane Yardley , and Richard Bracken . 2018. “Interstitial Glucose and Physical Exercise in Type 1 Diabetes: Integrative Physiology, Technology, and the Gap In‐Between.” Nutrients 10(1): 93. 10.3390/nu10010093. 29342932 PMC5793321

[ejsc12220-bib-0030] Muñoz Fabra, Elena , J.‐Luis Díez , Jorge Bondia , and Alejandro José Laguna Sanz . 2021. “A Comprehensive Review of Continuous Glucose Monitoring Accuracy during Exercise Periods.” Sensors 21(2): 479. 10.3390/s21020479. 33445438 PMC7828017

[ejsc12220-bib-0031] Naftchi, N. Eric . 1985. “Alterations of Neuroendocrine Functions in Spinal Cord Injury.” Peptides 6: 85–94. 10.1016/0196-9781(85)90015-4. 3931063

[ejsc12220-bib-0032] Olsson J. Swedish Elite Swimmers Blood Glucose Levels during Recovery: A Descriptive Study Using Continuous Glucose Monitoring Systems. 2017.

[ejsc12220-bib-0033] Ricci, Francesco , Felice Caprio , Alessandro Poscia , Francesco Valgimigli , Dimitri Messeri , Elena Lepori , Giorgio Dall’Oglio , Giuseppe Palleschi , and Danila Moscone . 2007. “Toward Continuous Glucose Monitoring with Planar Modified Biosensors and Microdialysis: Study of Temperature, Oxygen Dependence and In Vivo Experiment.” Biosensors and Bioelectronics 22(9–10): 2032–2039. 10.1016/j.bios.2006.08.041. 17000099

[ejsc12220-bib-0034] Shah, Viral N. , Stephanie N. DuBose , Zoey Li , Roy W. Beck , Anne L. Peters , Ruth S. Weinstock , Davida Kruger , et al. 2019. “Continuous Glucose Monitoring Profiles in Healthy Nondiabetic Participants: a Multicenter Prospective Study.” Journal of Clinical Endocrinology and Metabolism 104(10): 4356–4364. 10.1210/jc.2018-02763. 31127824 PMC7296129

[ejsc12220-bib-0035] Skroce, Kristina , Andrea Zignoli , Federico Y. Fontana , Felipe M. Maturana , David Lipman , Andrea Tryfonos , Michael C. Riddell , and Howard C. Zisser . 2024. “Real World Interstitial Glucose Profiles of a Large Cohort of Physically Active Men and Women.” Sensors 24(3): 744. 10.3390/s24030744. 38339464 PMC10857405

[ejsc12220-bib-0036] Sprague, J. E. , and A. M. Arbeláez . 2011. “Glucose Counterregulatory Responses to Hypoglycemia.” Pediatric Endocrinology Reviews 9(1): 463. 22783644 PMC3755377

[ejsc12220-bib-0037] Supersapiens Dashboard [Accessed March to May 2023]. Available from: https://dashboard.supersapiens.com.

[ejsc12220-bib-0038] Thomas, Felicity , Christopher G. Pretty , Matthew Signal , J. Geoffrey Chase , and J. G. Chase . 2017. “Accuracy and Performance of Continuous Glucose Monitors in Athletes.” Biomedical Signal Processing and Control 32(20): 124–129. 10.1016/j.ifacol.2015.10.105.

[ejsc12220-bib-0039] Training Peaks [Accessed March to May 2023]. Available from: https://www.trainingpeaks.com.

[ejsc12220-bib-0040] Tukey, J. W. 1977. Exploratory Data Analysis: Reading, MA.

[ejsc12220-bib-0041] Wasserman, D. 1995. “Regulation of Glucose Fluxes during Exercise in the Postabsorptive State.” Annual Review of Physiology 57(1): 191–218. 10.1146/annurev.physiol.57.1.191. 7778865

[ejsc12220-bib-0042] Zisser, Howard C. , Timothy S. Bailey , Sherwyn Schwartz , Robert E. Ratner , and Jonathan Wise . 2009. “Accuracy of the SEVEN® Continuous Glucose Monitoring System: Comparison with Frequently Sampled Venous Glucose Measurements.” Journal of Diabetes Science and Technology 3(5): 1146–1154. 10.1177/193229680900300519. 20144429 PMC2769895

