# Supplementary material for: Continuous glucose monitoring in para cyclists: An observational study
Source: Eur J Sport Sci. 2024 Nov 25;24(12):1809–19. doi: 10.1002/ejsc.12220 (PMC11621371; doi:10.1002/ejsc.12220)
Supplement: Supplementary file 1 — Table S1 [file EJSC-24-1809-s001.docx]

**Supplemental Table 1. Nutritional intake for all meals and at breakfast, lunch and dinner separately.**

|  | Total | Breakfast | Lunch | Dinner | *P*-value |
| --- | --- | --- | --- | --- | --- |
| Energy (kcal) | 2768 (2354-3784) | 552 (454-802) | 757 (577-951) | 844 (721-871) | 0.061 |
| Carbohydrate (g) | 373±130 | 79±26 | 81±35 | 84±22 | 0.885 |
| Carbohydrate (en%) | 47±4 | 53±8 | 43±13 | 42±10 | 0.139 |
| Mono and disaccharide (g) | 110 (76-192) | 27 (20-49) | 12 (5-31) | 13 (11-19) | **0.025^a^** |
| Protein (g) | 143 (109-151) | 20 (17-31) | 30 (22-42) | 42 (39-64) | **<0.001^ab^** |
| Protein (en%) | 17 (16-20) | 14 (12-16) | 15 (14-19) | 24 (17-30) | 0.061 |
| Fat (g) | 96 (84-125) | 20 (12-28) | 34 (21-39) | 27 (20-41) | 0.301 |
| Fat (en%) | 32±4 | 29±8 | 37±11 | 32±10 | 0.307 |

Data are presented as mean±SD or median (IQR). *P*-values of the main effect are presented. ^a^ Significant difference between breakfast and dinner. ^b^ significant difference between lunch and dinner.
